# Supplementary material for: Transcriptomic Insights into Mechanisms of Early Seed Maturation in the Garden Pea (Pisum sativum L.)
Source: Cells. 2020 Mar 23;9(3):779. doi: 10.3390/cells9030779 (PMC7140803; doi:10.3390/cells9030779)
Supplement: Supplementary file 1 [file cells-09-00779-s001.zip › Supplementary materials/FileS2.zip!/FileS1/Summary Sheets - Modules/0verview.html]

Spot Summary of Full transcriptomic landscape dataset


# Expression Module Reports

Expression module detection applies different criteria to define spot clusters of co-regulated metagenes:
overexpression and underexpression, mutual correlations between the metagenes,
k-means clustering and cluster detection in the distance map.  

- General report sheets provide overview maps of the modules detected, module-related heatmaps characterizing the
  expression profiles of the selected features in the series of samples, and individual module reports
  providing detailed information such as ranked gene list selected with the module and functional analysis in terms of
  gene set lists ranked for over-representation in the module.

- Profile sheets provide the module expression profiles on single sample and group resolution.

- Module relation reports contain the wTO correlation network and association of the groups to the modules.

- Chromosome plots visualize enrichment of chromosomal postitions (chromosome/band) in the modules as overview heatmaps and individual chromosome plots.

## Overexpression Spots

- General module report (PDF)
- Module Profiles (PDF)
- Module Relation (PDF)
- Module Enrichment in Chromosomes (PDF)

## Group Overexpression Spots

- General module report (PDF)
- Module Profiles (PDF)
- Module Relation (PDF)
- Module Enrichment in Chromosomes (PDF)

## Underexpression Spots

- General module report (PDF)

## Correlation Cluster

- General module report (PDF)

## K-Means Cluster

- General module report (PDF)
- Module Profiles (PDF)
- Module Relation (PDF)
- Module Enrichment in Chromosomes (PDF)

## D-Cluster

- General module report (PDF)
- Module Profiles (PDF)
- Module Relation (PDF)
- Module Enrichment in Chromosomes (PDF)

# Expression Module CSV Sheets

|  | CSV Tables |
| --- | --- |
| Overexpression Spots | AB |
| Group Overexpression Spots | ABC |
| K-Means Cluster | ABCDEFGHIJKLMNOPQRST |
| Distance Map Cluster | ABC |
